# Supplementary material for: Remediation of heavy metal contaminated soil by asymmetrical alternating current electrochemistry
Source: Nat Commun. 2019 Jun 4;10:2440. doi: 10.1038/s41467-019-10472-x (PMC6547649; doi:10.1038/s41467-019-10472-x)
Supplement: Supplementary file 1 — Supplementary Information [file 41467_2019_10472_MOESM1_ESM.pdf]

Supplementary Information

Remediation of heavy metal contaminated soil by asymmetrical alternating current electrochemistry

Xu et al.

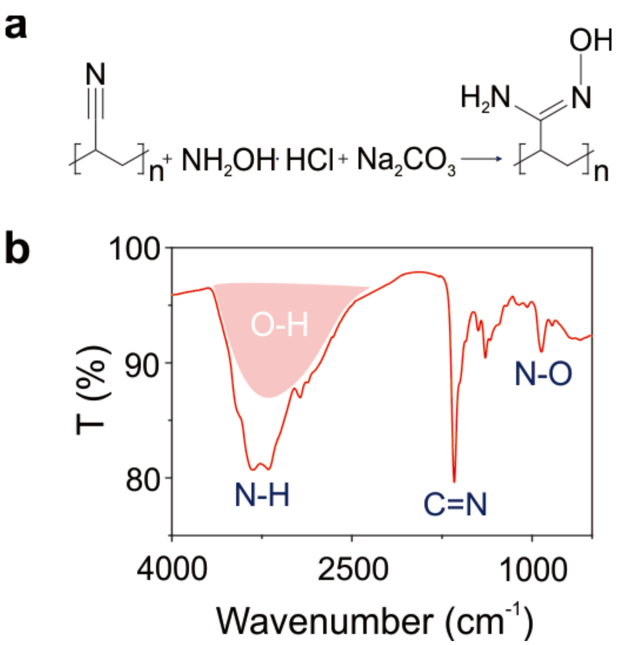

Supplementary Fig. 1. Synthesis and characterization of amidoxime. (**a**) Hydrothermal reaction that substitutes the nitrile functional groups in PAN with amidoxime functional groups. (**b**) Fourier transform infrared spectroscopy (FTIR) of the Ami-PC electrode confirming the existence of amidoxime.

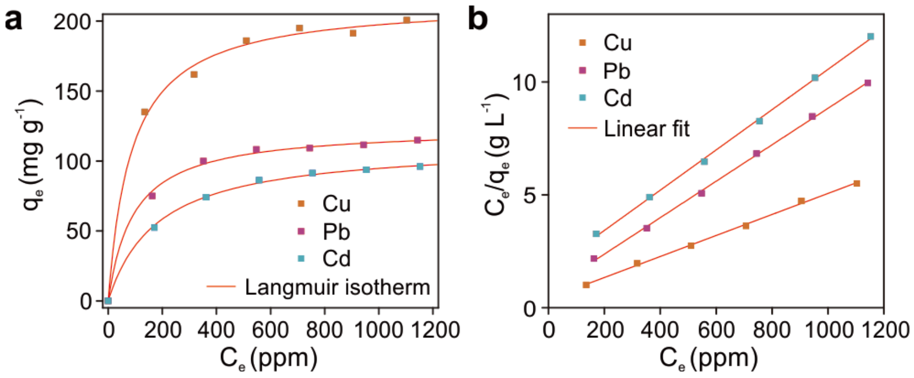

c

Adsorbate	q_m (mg g^{-1})	K_L (L mg^{-1})
Cu	214.6	0.0115
Pb	123.9	0.0106
Cd	112.1	0.0054

Supplementary Fig. 2. Adsorption isotherms of heavy metals on the Ami-PC electrode. (**a**) The equilibrium adsorption amounts (q_e) of Cu, Pb and Cd ions onto the Ami-PC electrode after the equilibrium time (6 h) as a function of equilibrium concentration (C_e) in the solution, fitted by the Langmuir model (equation 1). (**b**) The adsorption isotherms displayed in their linearized format (equation 2). (**c**) The Langmuir isotherm parameters are calculated from the slope and intercept of the linear fitting in b. The q_m and K_L represent the maximum adsorption capacity of the Ami-PC electrode and the Langmuir adsorption constant. The high adsorption capacity indicates the strong chelation sites provided by the amidoxime coating.

$$q_e = \frac{q_m K_L C_e}{1 + K_L C_e}$$ (equation 1)

$$\frac{C_e}{q_e} = \frac{1}{q_m K_L} + \frac{C_e}{q_m}$$ (equation 2)

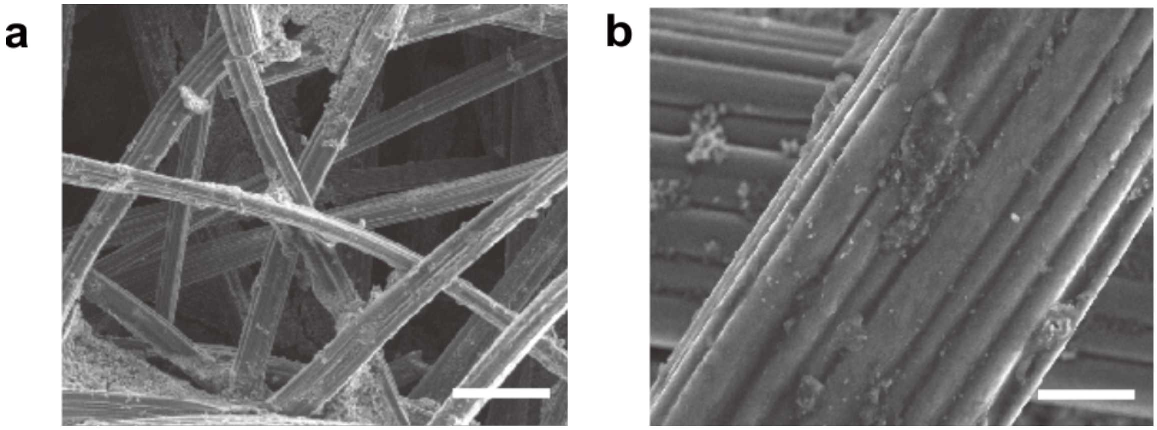

Supplementary Fig. 3. Morphology of the Ami-PC electrode. (**a**) SEM image showing the morphology of the Ami-PC electrode. Scale bar, 50 μm. (**b**) Magnified SEM image showing the uniform coating of amidoxime on the surface of carbon fibres. Scale bar, 5 μm.

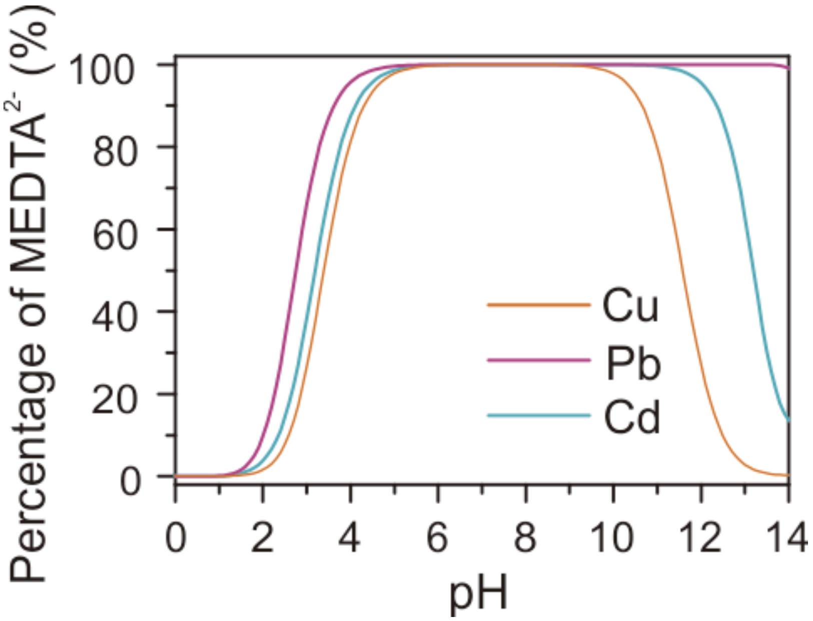

Supplementary Fig. 4. Speciation calculation using Visual MINTEQ. The figure shows the species distribution of 100 ppm heavy metal cations in a 30 mM EDTA solution. In a wide pH range, ~100% heavy metal cations exist as anionic complex ($MEDTA^{2-}$).

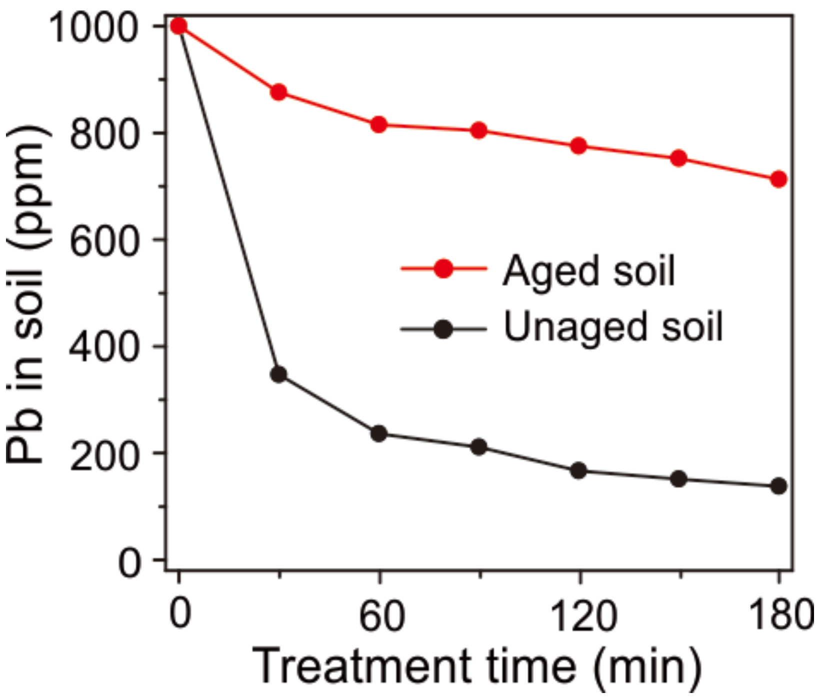

Supplementary Fig. 5. Washing the contaminated soil (1,000 ppm Pb) with deionized water. For the unaged soil (spiked with $Pb(NO_3)_2$ solution without posttreatment), washing with pure water can leach out a large proportion (~80%) of the spiked Pb. However, for the aged soil, pure water can only leach out 20% of the Pb after 3 hours, which is much less compared with our AACE method and the fresh EDTA solution wash (Fig. 2b). This result corroborates that the aging process enabled the heavy metals to adsorb to the soil particles and decreased their solubility and mobility.

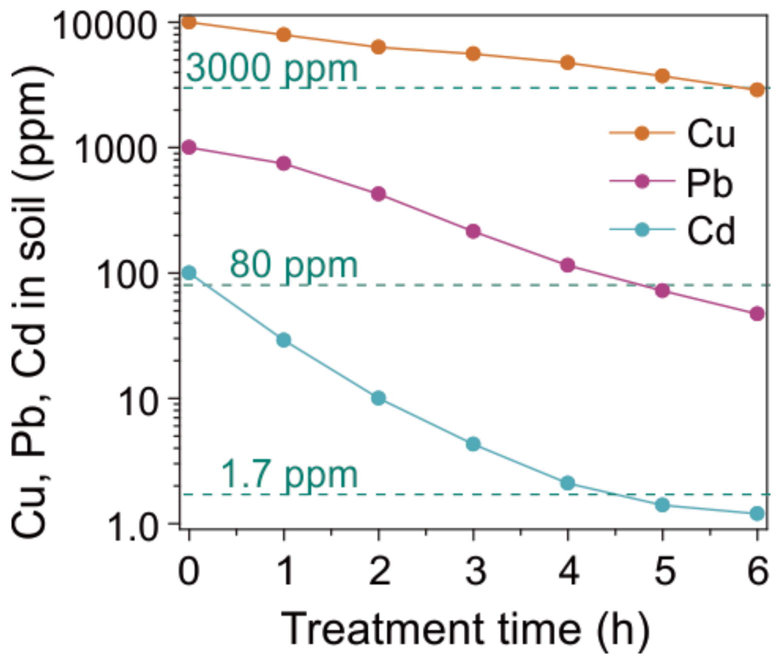

Supplementary Fig. 6. Remediation performance for mixed contamination. Concentrations of heavy metals in mixed contaminated soil during AACE remediation. Initial contaminant concentrations: Cu, 10,000 ppm; Pb, 1,000 ppm; Cd, 100 ppm.

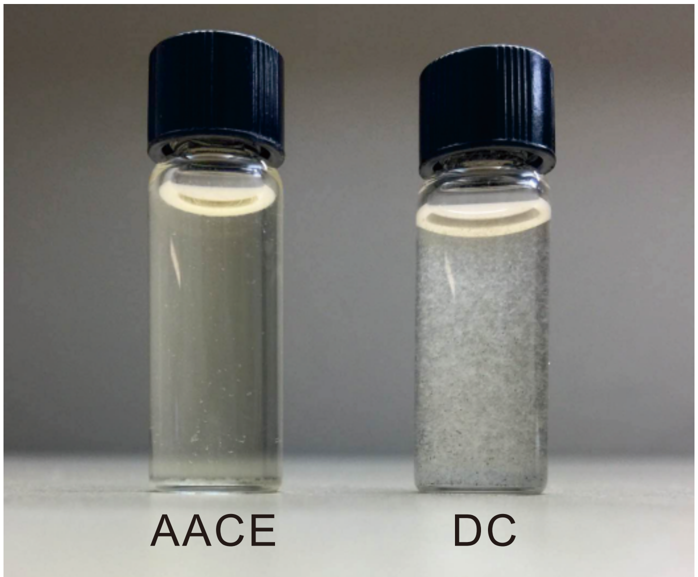

Supplementary Fig. 7. Washing effluent after electrochemical filtration. Left, washing effluent after AACE filtration has a clear pale yellow colour. Right, flocculent precipitate in the washing effluent after DC filtration.

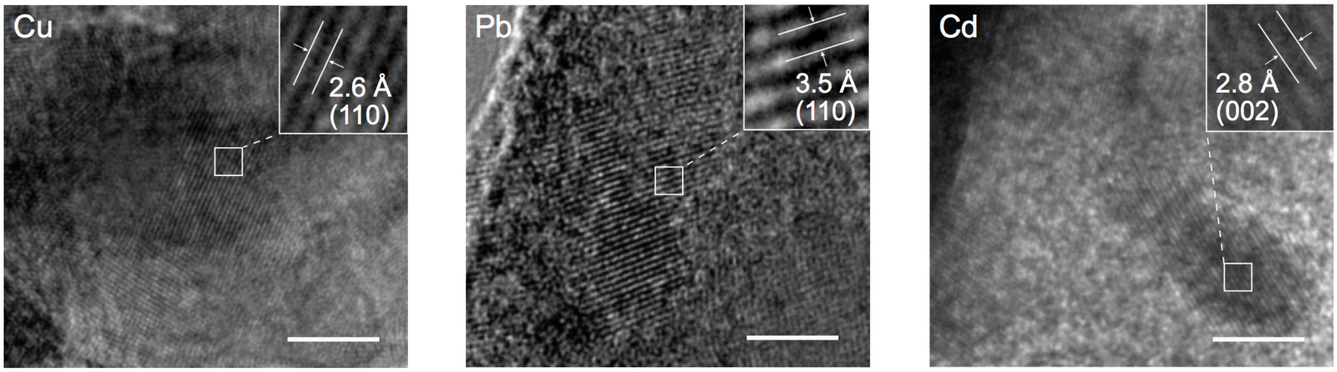

Supplementary Fig. 8. Crystal structures of the extracted heavy metal particles. TEM images showing the crystal structures of Cu, Pb and Cd particles extracted by the AACE method. Scale bars, 5 nm. Insets show magnified view of lattice spacing.

a b

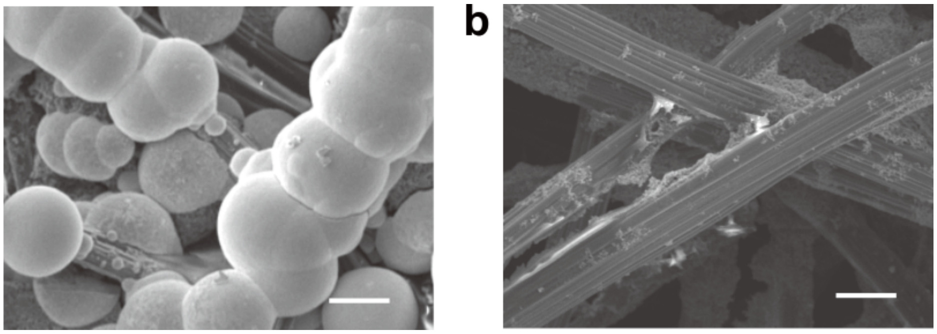

Supplementary Fig. 9. Regeneration of the AACE filter. (**a**) SEM image showing the morphology of the Ami-PC electrode after long-term AACE filtration. Scale bar, 20 μm. (**b**) SEM image of the regenerated Ami-PC electrode after acid elusion. Scale bar, 20 μm.

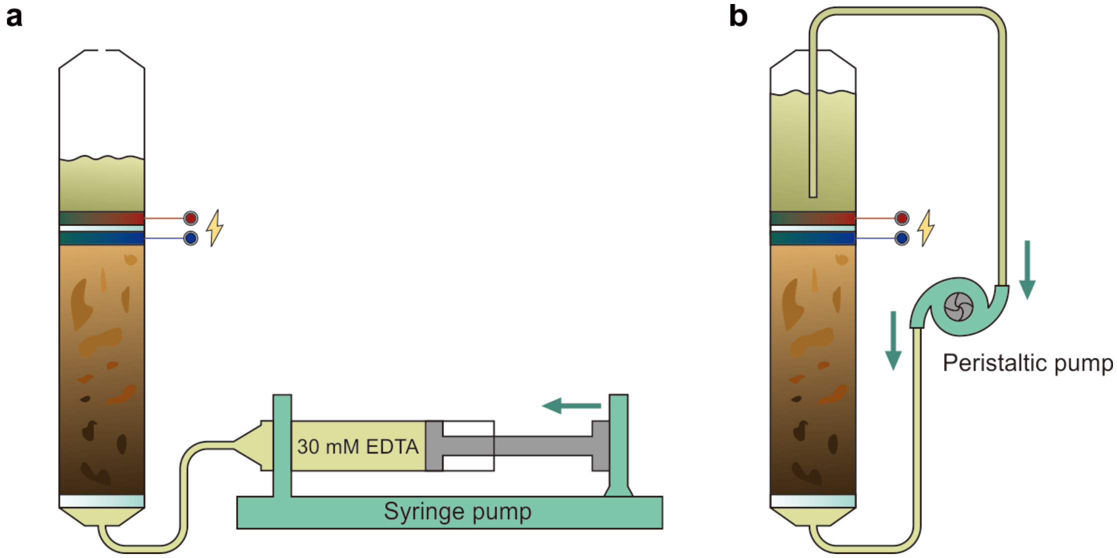

Supplementary Fig. 10. Illustration of the experimental setup. (**a**) A syringe pump was first used to infuse the soil washing solution, which washed through the soil column and the AACE filter, and accumulated above the AACE filter. (**b**) After all the soil washing solution went through the soil column for the first time, a peristaltic pump was used to circulate the solution to wash through the soil column repeatedly.

Supplementary Table 1. Textural analysis and physicochemical properties of the fresh and aged soil. OC, organic carbon. CEC, cation exchange capacity.

	Sand (%)	Silt (%)	Clay (%)	pH	OC (g kg^{-1})	CEC ($cmol_c$ kg^{-1})
Fresh soil	45	37	18	6.2	28.4	17.9
Aged soil	45	39	16	6.8	25.5	18.3
